# Nutritional optimization of fecal microbiota transplantation in humans: a scoping review

**DOI:** 10.1080/19490976.2024.2446378

**Published:** 2025-01-08

**Authors:** Levi M Teigen, Austin Hoeg, Hijab Zehra, Priyali Shah, Remy Johnson, Kristen Hutchison, Megan Kocher, Annie W Lin, Abigail J Johnson, Byron P Vaughn

**Affiliations:** a Department of Food Science and Nutrition, University of Minnesota, St. Paul, MN, USA; b Medical School, University of Minnesota, Minneapolis, MN, USA; c Achieving Cures Together, St. Louis Park, MN, USA; d University of Minnesota Libraries, St. Paul, MN, USA; e The Hormel Institute, University of Minnesota, Austin, MN, USA; f Division of Epidemiology and Community Health, School of Public Health, University of Minnesota, Minneapolis, MN, USA

**Keywords:** Fecal microbiota transplantation, diet, microbiome, nutrition, dietetics

## Abstract

Diet constitutes a major source of nutrient flow to the gut microbes. As such, it can be used to help shape the gut microbiome. Fecal microbiota transplantation (FMT) is an increasingly promising therapy in disease states beyond recurrent *Clostridioides difficile* infection, but diet is largely overlooked for its potential to help optimize this therapy. Therefore, the aim of this scoping review is to present the literature landscape that captures pre- and post-FMT dietary intake in humans, identify research gaps, and provide recommendations for future research. A comprehensive search strategy was developed and searches were run in five databases. Studies were included if they discussed adults who underwent FMT for any recognized treatment indication and had dietary intake as a study objective, this search encompassed studies with interventions that included foods and dietary supplements. The initial screening identified a total of 7721 articles, of which 18 met the inclusion criteria for this review. Studies were heterogeneous, but taken together, they introduce a framework that defines important nutritional considerations for both donors and FMT recipients in the period around FMT dosing. This framework is summarized with this review and highlights the opportunities available to develop FMT-based precision nutrition strategies to optimize its clinical efficacy.

## Introduction

The human gut microbiome is a complex internal ecosystem that is exposed to the external environment by its location within the gastrointestinal tract.^1^ It is shaped by both host and environmental variables,^2^ but most of these either cannot be, or are not, easily changed. To date, the most dynamic microbiome-targeted therapy is fecal microbiota transplantation (FMT), which can successfully impact several disease states.^3^ Diet is an important modifiable variable that contributes to shaping the gut microbiome, but the extent to which diet impacts the success of FMT is not known.

FMT is premised on the correction of dysbiosis in recipients and seeks to restore microbial homeostasis through transplantation of a healthy fecal microbiota community from a stool donor. The therapy is highly effective to prevent recurrent *C. difficile* infection (rCDI) and is increasingly being studied in other clinical conditions.^3^ FMT represents a live therapeutic shaped by donor diet-derived nutrient flow to the gut microbes that, after transplant, becomes susceptible to recipient diet and nutrient flow. Therefore, diet in both the pre- and post-FMT periods should be considered to optimize success of this therapy.

### Mechanistic framework for approaching nutritional optimization of FMT

Understanding the complementary role of diet to FMT requires defining what is considered “success” of FMT (e.g. engraftment, microbial function, or clinical improvement). For example, if the desired endpoint of FMT is engraftment, then the goal of diet therapy would be to sustain donor specific taxa (i.e., compositional support). Alternatively, if the desired endpoint of FMT is related to functional capacity of the microbes, such as increased short chain fatty acid production, then the goal of diet therapy would be to support the metabolic potential of the transplanted microbes (i.e., functional support). While FMT therapy is premised on a correction of an underlying dysbiosis, it is also possible that diet can support clinical endpoint success of FMT therapy through indirect mechanisms, such as correction of malnutrition,^4^ immune support, or complementary management of systemic metabolic endpoints^5^ (i.e., clinical support).

#### Compositional support

The composition of the human gut microbiome is largely resistant to short-term diet changes but can be susceptible to fiber free interventions (e.g., exclusive enteral nutrition) within 5 days. These findings suggest that even short-term periods of fiber-free intake (e.g., NPO, fiber-free enteral nutrition) by the recipient may negatively impact FMT success, particularly in the immediate post-FMT period. By contrast, donor microbiota are largely shaped by long-term dietary intake, which can influence the presence of specific taxa relevant to the underlying disease being treated with FMT. Animal models have the capacity for an irreversible loss of microbes over several generations with the removal of fiber from the diet^6^; similar findings are observed across generations in humans with Western-style diets. Knowledge of donor dietary intake, therefore, could inform recommendations for the recipient post-FMT diet most likely to support the microbes of interest (i.e., recipients aim to consume a diet similar to a donor’s habitual diet). Alternatively, identifying donors who harbor the taxa of interest and consume a pre-FMT diet similar to recipients could be used to facilitate success of compositional FMT endpoints without the need for post-FMT recipient diet changes. This approach will require knowledge of both donor and recipient baseline diet.

#### Functional support

Diet’s most significant impact on the gut microbiome may be its effects on function, which can occur independent of compositional changes.^7^ While the exact contribution of diet to compositional variation within the gut microbiota is unknown (estimated <20% but some estimates > 50%),^2,8–10^ the effect of diet on function is likely more consistent.^11^ While some functions are specific to certain microbes (e.g. methanogenesis and methanogens), functional redundancy – presence of distinct microbial species with the same metabolic function – often results in a microbial metabolic flexibility that is highly responsive to nutrient flow. What this means in practice is that microbial functional characteristics observed in the donor (e.g., production of a beneficial metabolite or limited production of a harmful metabolite) may largely be the result of dietary intake and, without a corresponding diet in the recipient, fail to transplant as part of the FMT. Similar to compositional support, knowledge of pre-FMT dietary intake of both the donor and recipient can help match donors who harbor microbial functions of interest with recipients whose diets will sustain that function. Alternatively, knowledge of donor dietary intake can be used to inform post-FMT diet recommendations for recipients in order to support the microbial function of interest.

#### Clinical support

Although categorized as a drug by the FDA, conceptually, FMT is more akin to organ transplantation. Like transplant and surgery, perioperative nutrition, particularly undernutrition, may play an important role in improving outcomes. Malnutrition is highly prevalent in clinical populations receiving FMT, such as hospitalized patients, patients with inflammatory bowel disease (IBD), and patients with rCDI.^12,13^ Pre-operative malnutrition is associated with poor clinical outcomes and delayed recovery in surgical populations^14^ and may also impact success of FMT. In clinical populations with obesity and metabolic syndrome, diet has been shown to independently improve disease parameters; and, as exemplified by medications used to manage features of metabolic syndrome, diet is often intended to be used as an adjunct to optimize medication therapy.^15^ Therefore, in addition to the direct effect of diet on the transplanted microbes, nutrition may also play a broader role in support of clinical FMT endpoints.

**Figure 1.**
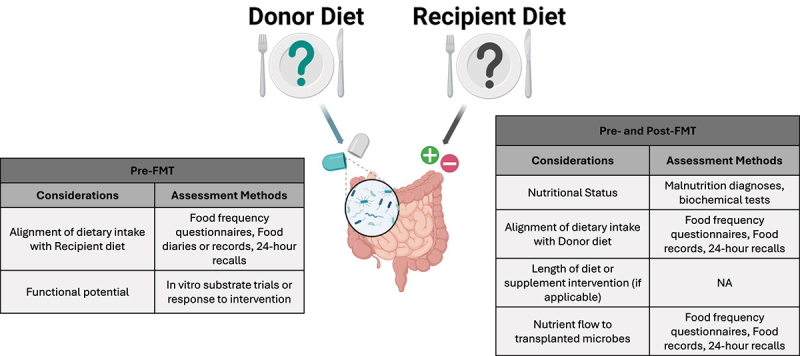
Nutritional considerations associated with fecal microbiota transplant (FMT) therapy. It is important to consider both donor and recipient dietary intake as well as nutritional status of the recipient in the pre-FMT period. There are also important nutritional recipient considerations in the post-FMT period.

The mechanisms through which FMT ultimately impacts clinical endpoints are incompletely understood. Consequently, the role of nutrition therapy should be approached through a comprehensive framework that is applicable across disease states and potential FMT mechanisms. As outlined in Figure 1, these dietary intake considerations extend from the pre-FMT intake of both donor and recipient to post-FMT intake of the recipient. The aim of this scoping review is to present the literature landscape that captures pre- and/or post-FMT dietary intake in humans to identify research gaps and enhance future study designs to further our understanding of dietary management of FMT.

## Methods

The objectives, inclusion criteria, and methods for this scoping review were prespecified and published in a protocol with Open Science Framework.^16,17^ We used previously established scoping review methodology to guide our study methods and applied the Preferred Reporting Items for Systematic Reviews and Meta-Analyses for Scoping Reviews (PRISMA-Scr).^16^

### Search strategy, literature sources, & supplementary data

A comprehensive search strategy was developed and run by a science librarian (MK) in collaboration with subject matter experts (LT and AH) and tested against a set of exemplar articles. Searches were run in five databases: Medline via Ovid, Agricola via Ovid, Embase Classic + Embase via Ovid, Scopus via Elsevier, and the Cochrane Library via Wiley. No date or language limits were applied in the databases. Limits were applied to remove Medline results from databases other than Medline. Searches related to fecal microbiota transplantation and dietary intake terms were run on May 13, 2024. Full search strategies are available in Appendix A.

### Inclusion criteria

Inclusion criteria were defined based on the Patient, Concept, and Context criteria outlined by the Joanna Briggs Institute methodology for scoping reviews: any papers directly discussing adults who underwent FMT for any recognized treatment indication with dietary intake as a study objective, encompassing relevant food, supplements, and supplemented foods.^16^ As per Effective Practice and Organization of Care reviews criteria, the following study designs were considered for inclusion: randomized control trials, non-randomized control trials, controlled before-after studies, and interrupted time series. Case reports and case series were also considered for inclusion. Non-English publications presented with an English language abstract were included if they had sufficient evidence for extraction.^18^ Of note, both quantitative and qualitative studies from each of these categories were included. Reviews (e.g., meta-analyses, systematic reviews, narrative reviews), chapters and books, gray literature (dissertations, reports, conference proceedings), unpublished, non-peer-reviewed articles, and all other non-original articles were excluded.

**Figure 2.**
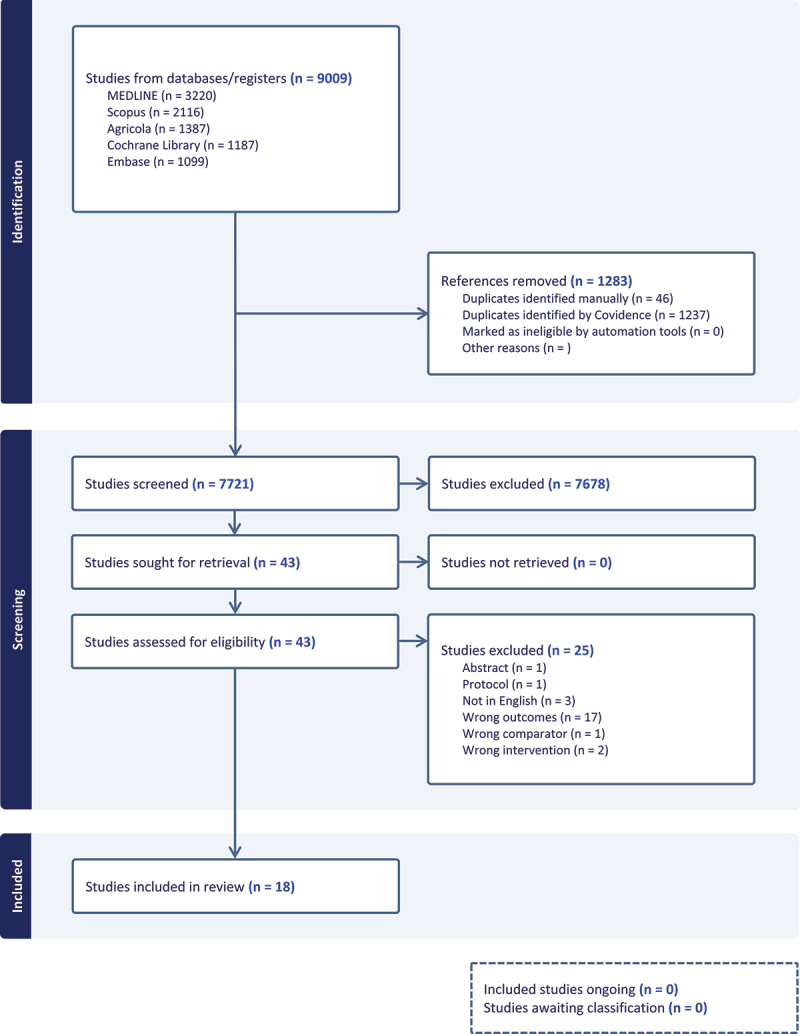
PRISMA flow diagram outlining article selection process.

Results from the literature search (Figure 2) were uploaded to Covidence, a web-based collaboration software platform that streamlines the production of systematic and other types of literature reviews.^19^ Duplicate references were removed to the extent possible, and the articles were screened for eligibility based on the inclusion and exclusion criteria prior to data extraction.

The abstract stage inclusion criteria were any study that discussed some variation of the term fecal microbiome transplant and diet or supplement. Article titles and abstracts were independently reviewed for eligibility by authors. Titles and abstracts must have discussed FMT and included language suggesting they were interested in dietary or supplement intake as a study objective while reporting on dietary intake in their findings section. Once references were determined to be potentially eligible, full articles were obtained for studies that appeared to meet the eligibility criteria based on title and abstract screening. Next, the full text was independently reviewed by two coders for eligibility and inclusion. Five authors (LT, AH, HZ, PS, and RJ) assumed this role and completed the title/abstract and full-text screening. At both the abstract and full-text stages, disagreement was resolved by bringing the first author (LT) in for discussion if required.

### Data extraction, synthesis, & analysis

A charting table was developed a priori by two reviewers (LT and AH). The charting table consisted of predefined data items for extraction, which included title, authors, publication year, age, sex, race/ethnicity, the condition being treated, type of donor material (e.g., single, combined), transplant approach (e.g., colonoscopic, oral capsules), number of transplants, dietary intervention, any microbiome-related dietary intake data, and demographic and study characteristics. The charting form was revised iteratively, as needed while screening each of the included articles. Four reviewers (AH, HZ, PS, and RJ) extracted data from the original search; a final reviewer then independently verified the data (LT). Any discrepancies were resolved by re-review of the study or discussion with the final reviewer (LT).

### Patient & public involvement

No patients were involved.

## Results

We found 7721 relevant studies, of which 43 met the inclusion criteria for screening (Figure 2). After a full-text review, 16 original manuscripts were included in this scoping review, along with two secondary analyses^20,21^ for a total of 18 papers (Table 1). The conditions studied included: rCDI,^22^ irritable bowel syndrome (IBS) both constipation and diarrhea phenotypes,^23–26^ cardiovascular disease,^27^ inflammatory bowel disease (IBD) both ulcerative colitis (UC)^20,28–31^ and Crohn’s disease (CD)^32^ as well as chronic pouchitis,^33^ weight loss,^34^ and metabolic syndrome,^21,35,36^ and type 2 diabetes mellitus.^37^

**Table 1.** Overview of all studies included.

Author	Year	Location	Design	Condition being treated	n	Mean age, years	Intervention	Single vs. Combined Donor Material	Pre-FMT Donor diet data	Pre-FMT Recipient diet data	Post-FMT Recipient diet data	Diet Assessment Method
Ge et al. ^24^	2016	China	Prospective, Open-label	Slow transit constipation	21	50	Fiber supplementation: Pectin, 8 g twice daily for 4 weeks	Combined	-	-	+	NA
Niccum et al. ^22^	2018	United States	Retrospective	rCDI	80	66	None	NA	-	-	-	NA
Smits et al. ^27^	2018	The Netherlands	Double-blind, RCT	Metabolic syndrome	20	55	FMT from vegan donor or Autologous	Single	+	-	-	7-day dietary record
Zhang et al. ^26^	2018	China	Prospective, Open-label	Slow transit constipation	31	42	Fiber supplementation: Group 1: FMT+highly fermentable fiber Group 2: FMT+low fermentable fiber Group 3: Placebo FMT+highly fermentable fiber Group 4: Placebo FMT+low fermentable fiber	Single	-	-	+	NA
Costello et al. ^29^	2020	Australia	Case report	Ulcerative colitis	1	19	Dietary intervention: Increased fiber, reduced sulfate intake	Single	-	-	+	NA
Kousgaard et al. ^33^	2020	Denmark	Observational	Chronic pouchitis	9	52	None	Combined	-	-	-	NA
Bryant et al. ^28^	2021	Australia	Case Report	Ulcerative colitis	1	71	Dietary intervention: 4-SURE diet (similar to Costello et al.)	Single	-	-	+	7-d diet record at baseline and 3-d diet record at weeks 4, 8, and 24
Clancy et al. ^23^	2021	Australia	Observational	Irritable bowel syndrome or Inflammatory bowel disease	18	35 (averaged median)	None	Combined	-	-	-	3-d diet records
Koopen et al. ^35^	2021	The Netherlands	Double-blind, RCTl	Obesity with Metabolic Syndrome	24	52	Mediterranean diet for 2-weeks pre-FMT. Continued for 6-weeks post-FMT	Single	+ (autologous FMT) and - (donor FMT)	+	+	48-h diet record on 3 separate occasions
Mocanu et al. ^36^	2021	Canada	Four-arm, parallel, placebo controlled, RCT Randomized 1:1:1:1 and stratified by sex	Obesity (BMI >30) with Metabolic syndrome	70	48	Fiber supplementation: Group 1: FMT+highly fermentable fiber Group 2: FMT+low fermentable fiber Group 3: Placebo FMT+highly fermentable fiber Group 4: Placebo FMT+low fermentable fiber	Combined	-	-	+	Food frequency questionnaire
Rinott et al. ^34^	2021	Israel	Randomized, open-label	Abdominal obesity	90	52	6-month intervention pre-FMT. Group 1: Healthy dietary guidelines Group 2: Mediterranean Diet Group 3: Green Mediterranean Diet	Single	-	-	+	Food-frequency questionnaire
Xiang et. al. ^32^	2021	China	Randomized, prospective, open-label	Crohn’s Disease with Malnutrition	19	37.5 (averaged median)	Exclusive enteral nutrition (EEN) intervention for 15 days + FMT on either day 1 or 8	Combined	-	-	+	NA
Huang et al. ^25^	2022	China	Two-arm, open-label, prospective	Refractory IBS-D	80	41	FMT or FMT+Diet Diet intervention: Low FODMAP diet	Combined	-	-	+	Food diary
Kedia et al. ^30^	2022	India	Open-label, RCT	Ulcerative colitis	73	36	FMT+AID or Standard medical therapy	Combined	-	-	+	7-d diet records or Food frequency questionnaire
Shabat et al. ^31^	2022	Israel	Three-arm, single-blinded, RCT Randomized 1:1:1	Ulcerative Colitis	62	40	Group 1: FMT+Free diet Group 2: FMT+UCED diet Group 3: UCED diet	Combined	-	-	+	NA
Su et al. ^37^	2022	China	Open-label, RCT	Type 2 Diabetes	16	59	Fiber supplementation: FMT or FMT+Supplement Supplement: Probiotic, prebiotic, whole grain (PPW) mixture	Combined	-	-	+	NA
*Leibovitz et al. ^20^	2024	Israel	*Post hoc analysis of Shabat et al. 2022^31^									
*Zhang et al. ^21^	2024	Canada	*Post hoc analysis of Mocanu et al. 2021^36^									

RCT = Randomized controlled trial; rCDI = recurrence *Clostridioides difficile* infection; NA = not available.

Studies were heterogeneous in their design with a combination of observational and interventional studies, as well as randomization and blinding. In addition, FMT preparation and administration, diet assessment tools, and outcomes varied between studies. Studies introduced relevant nutritional considerations for both donors and FMT recipients in the period around FMT dosing. Eight studies addressed pre-FMT intake of both donors and recipients, which included both donor and recipient dietary intake as well as pre-FMT nutrition status (e.g., presence of malnutrition and serum zinc status) of the recipients. Twelve post-FMT dietary intake studies included supplementation and diet interventions post-FMT. Overall, the available studies provide a comprehensive overview of fundamental nutritional considerations with FMT (Figure 1).

### Pre-FMT dietary intake

Eight studies reported data on pre-FMT dietary intake of either the donor or recipients (Table 2).^20–22,25,27,32,33,35^ A ninth study, using autologous FMT with diet pre-conditioning, was also included in this section.^34^

**Table 2.** Overview studies assessing pre-FMT dietary intake.

Author	Year	Location	Design	Condition being treated	Intervention	Donor diet	Diet assessment method	Comments
Niccum et al. ^22^	2018	United States	Retrospective	Recurrent *Clostridioides difficile* infection	None	NA	NA	Zinc status assessed by serum zinc measures
Smits et al. ^27^	2018	The Netherlands	Double-blind, RCT	Metabolic syndrome	FMT from vegan donor or Autologous	Vegan diet	7-d diet records	Pre-FMT calorie and macronutrient intake similar between donor and recipient. Fiber intake ~ 43 grams/day in the vegan donors and ~18 g/day in recipients.
Kousgaard et al. ^33^	2020	Denmark	Observational	Chronic pouchitis	None	Food frequency questionnaire	NA	Past month food frequency questionnaires were used. Pre-FMT intake of yogurt associated with remission after FMT
Koopen et al. ^35^	2021	The Netherlands	Double-blind, RCT	Obesity with Metabolic Syndrome	Mediterranean diet for 2-weeks pre-FMT. Continued for 6-weeks post-FMT.	NA	48-h diet record on 3 separate occasions	Subjects were provided food boxes suited for two adults to facilitate adherence Mediterranean diet intervention decreased calorie, saturated fat, and carbohydrate intake, and increased fiber intake from 20 to 24 g/day. In the context of decreased calorie intake, this translated to a ~ 45% increase in fiber intake from ~9 g/1000 kcal at baseline to ~13 g/1000 kcal on diet.
Rinott et al. ^34^	2021	Israel	Randomized, open-label	Abdominal obesity (defined by waist circumference or dyslipidemia)	6-month intervention pre-FMT. Group 1: Healthy dietary guidelines Group 2: Mediterranean Diet Group 3: Green Mediterranean Diet	NA	Food frequencyquestionnaire	Participants who lost >/ = 3.5% body weight on the intervention at 6 months recruited to a double-blind, placebo-controlled autologous FMT study from 6–14 months Microbiome composition changes only observed in Group 3 during the 6-month pre-FMT intervention.
Xiang et. al. ^32^	2021	China	Randomized, prospective, open-label	Crohn’s Disease with Malnutrition	Exclusive enteral nutrition (EEN) intervention for 15 days + FMT on either day 1 or 8	NA	NA	Parenteral nutrition was used to achieve caloric intake of at least 30 kcal/kg/day EEN formula or amount of EEN not provided. Malnutrition diagnosed according to Patient-Generated Subjective Global Assessment
Huang et al. ^25^	2022	China	Two-arm, open-label, prospective	Refractory IBS-D	FMT or FMT+Diet Diet intervention: Low FODMAP diet	No fast food or alcohol^38^	Food diary	No dietary intake data provided. Only noted that participants were “required to maintain food diary”. Enrollment in FMT+Diet arm based on “willingness to follow a low FODMAP diet”.
*Leibovitz et al. ^20^	2024	Israel	*Post hoc analysis of Shabat et al. 2022^31^	Ulcerative Colitis	Group 1: FMT+Free diet Group 2: FMT+UCED diet Group 3: UCED diet	Group 1: NA Group 2: 2-week conditioning with UCED diet prior to donating	NA	Diet adherence assessed, but methods not provided Dietary intake data not collected
Zhang et al. ^21^	2024	Canada	*Post hoc analysis of Mocanu et al. 2021^36^	Obesity (BMI >30) with Metabolic syndrome	Fiber supplementation: Group 1: FMT+highly fermentable fiber Group 2: FMT+low fermentable fiber Group 3: Placebo FMT+highly fermentable fiber Group 4: Placebo FMT+low fermentable fiber	NA	Food frequency questionnaire	Increased alpha-diversity following FMT was positively associated with plant protein, total and refined grains, and iron, thiamine, and folate. Intake of soluble fiber, sugars, and total carbohydrates were associated with engraftment. Defined according to change in insulin sensitivity from baseline to week 6, soluble fiber intake was associated with engraftment in “responders” and sugars and carbohydrates were associated with engraftment in “non-responders”.

RCT = Randomized controlled trial; NA = Not available; FODMAP = Fermentable Oligo-, Di-, Mono-saccharides, and Polyols; IBS-D = Irritable Bowel Syndrome with Diarrhea; UCED = Ulcerative Colitis Exclusion Diet.

#### Dietary intake of stool donors

Two studies limited donor selection to qualified individuals already following defined diets,^25,27^ and a third study used a 2-week dietary intervention as a conditioning period for donors.^20^ The first study provided minimal information about donor diet by stating that donors had “good dietary habits and healthy lifestyles.”^25^ The authors provided a citation that defined good dietary habits as avoidance of fast food and alcohol.^38^

Smits et al.^27^ highlighted the need to understand the *functional potential* of transplanted microbes and possible differences in donor and recipient dietary intake. In this study, investigators specifically recruited vegan donors to assess the impact of FMT from vegan donors on trimethylamine-N-oxide (TMAO) production and vascular inflammation in patients with metabolic syndrome. In total, nine vegan stool donors participated in the study. Vegan donors had an average age of 33.4 ± 14.8 years old with a mean BMI of 22.9 ± 1.7 kg/m^2^. The dietary intake of donors and recipients was assessed with a 7-day online food diary. Mean calorie intake did not differ between donors and recipients (~2,000 kcal/day). Macronutrient intake was also similar between the two groups. Fiber intake, however, was 43.4 ± 19.2 grams/day in the vegan donors and only 18 ± 4.2 grams/day in recipients. The vegan donor FMT product successfully altered fecal microbiota composition in recipients, but this result did not translate to reduced TMAO production.

The last study,^20^ a secondary analysis of the original CRAFT UC trial,^31^ lends additional support to alignment of donor intake with recipient intake post-FMT. The CRAFT UC trial included three groups of patients with mild to moderate ulcerative colitis. One of the groups (Group 2; FMT+diet intervention) received “diet conditioned” FMT where donor stool was collected after stool donors completed a 2-week dietary conditioning period. The donor dietary guidelines (Shabat et al.^31^; Supplementary Table S2), while not explicitly stated, seemed to align with the recipient ulcerative colitis exclusion diet (UCED) guidelines. Unfortunately, no dietary data are provided for either recipients or donors. Seven donors were included in a pre/post-dietary preconditioning analysis. Supporting the effect of short-term dietary intake on shaping the microbiome, the 2-week dietary preconditioning led to a change in the relative abundance of 51 species and the depletion of nine microbial metabolic pathways. It is unknown how different the 2-week preconditioning diet was from the donors’ baseline diets. Ultimately, post-FMT, the patients in Group 2 (diet conditioned FMT+UCED diet; i.e., post-FMT recipient diet aligned with donor diet) experienced an increase in alpha diversity and a shift toward donor microbial composition that was not observed in Group 1 where donor stool was not diet conditioned and recipients followed their regular diet.

#### Dietary intake of recipients pre-FMT only

Five studies were identified that only discussed pre-FMT recipient dietary intake.^21,22,32,33,35^ Three studies identified dietary intake variables that predicted clinical endpoints or engraftment,^21,22,33^ another study used a 2-week Mediterranean diet intervention prior to FMT for treatment of metabolic syndrome,^35^ and a final study used a 1-week exclusive enteral nutrition (EEN) intervention prior to FMT in a cohort of patients with malnutrition and active Crohn’s disease.^32^

Two observational studies assessed the impact of pre-FMT recipient nutrition status and dietary intake on clinical endpoints in rCDI and chronic pouchitis, respectively.^22,33^ The first, in rCDI, was a retrospective study that assessed pre-FMT zinc status within 90 days.^22^ Patients were categorized as either zinc deficient or replete and those found to be deficient were further categorized as to whether they received zinc supplementation. Investigators found that zinc deficiency increased the risk for CDI recurrence following FMT and that zinc supplementation reduced the risk. The second study was conducted in patients with chronic pouchitis.^33^ Dietary intake during the prior 6 months was collected through a food frequency questionnaire. Investigators found that increased yogurt consumption (mean 1.1 ± 0.6 servings/day vs 0.2 ± 0.4 servings/day) was associated with remission post-FMT. Regarding engraftment, a study aimed at determining the effect of FMT+fiber on metabolic outcomes in patients with obesity and metabolic syndrome found increased alpha-diversity following FMT was positively associated with plant protein, total and refined grains, and iron, thiamine, and folate.^21^ Investigators also found baseline intake of soluble fiber, sugars, and total carbohydrates to be associated with engraftment. Where responder status was defined according to change in insulin sensitivity from baseline to Week 6, soluble fiber intake was associated with engraftment in “responders,” and sugars and carbohydrates were associated with engraftment in “non-responders”.

An intervention study targeting insulin sensitivity in metabolic syndrome included a 2-week run-in period on a Mediterranean diet before FMT, followed by an additional 6 weeks on a Mediterranean diet.^35^ 48-hour diet records were used to measure dietary intake. The initial 2-week run-in period on the Mediterranean diet (pre-FMT) led to reductions in body weight, insulin resistance, and lipid levels. No additional effect was observed over the 6 weeks post-FMT. FMT was either from lean donors or autologous. No dietary intake data was available for the FMT donors; however, implementation of the Mediterranean diet led to decreased calorie intake, saturated fat, and carbohydrate intake, and ~ 45% increase in fiber intake (~9 g/1000 kcal at baseline and ~13 g/1000 kcal on diet) in recipients. The run-in period on the Mediterranean diet increased the abundance of *Bacteroides* species, *Akkermansia muciniphila*, *Roseburia hominis*, and reduced *Collinsella aerofaciens*.

Patients with inflammatory conditions such as IBD or rCDI undergoing FMT have a high likelihood of being malnourished.^12,39,40^ Therefore, it is important to understand what impact malnutrition might have on the success of FMT or, conversely, how FMT might support nutritional recovery in a patient who is malnourished. In a study of patients with both malnutrition and active Crohn’s flare, all participants received EEN for 15 days and were further randomized 1:1 to undergo FMT on day 1 or day 8.^32^ Patients were diagnosed with malnutrition based on the Patient-Generated Subjective Global Assessment. Nutritional parameters used by investigators were hemoglobin, lymphocyte count, albumin, and prealbumin. EEN with FMT on day 1 resulted in an improvement in albumin and prealbumin by day 8, but this improvement was not observed in the EEN group set to receive FMT on day 8. Metrics such as albumin and prealbumin are not considered meaningful markers of nutrition status and are indicative of active inflammation,^41^ but may still offer superficial insights into nutritional status.^42^ Clinically, the rate of clinical remission at day 15 (>70%) did not differ between groups.

#### Donor and recipient pre-FMT intake

A single study explored the effect of diet-modulated autologous FMT on weight regain in an obese cohort.^34^ Participants were assigned to one of three weight loss interventions for 6-months. The diets were 1) a Healthy Dietary Guidelines diet that consisted of “standard nutritional counseling to promote a healthy diet”; 2) a calorie-restricted (1500–1800 kcal/day for men and 1200–1400 kcal/day for women) Mediterranean Diet supplemented with 28 g/day walnuts; and 3) a Green-Mediterranean diet, which was similar to the Mediterranean diet but emphasized less red and processed meat and included an additional 4 cups/d green tea and 100 g *Wolffia globosa* duckweed. If target weight loss (≥3.5% body weight) was achieved at 6 months, participants transitioned into a double-blind, placebo-controlled autologous FMT trial. Beyond descriptions of the diet interventions, no information was provided on calorie and nutrient intake.

### Post-FMT dietary intake

Twelve studies were identified that targeted post-FMT dietary intake (Table 3). Four of these used fiber supplementation,^24,26,36,37^ and five of these used post-FMT diet interventions.^25,30–32,35^ A single study was an observational pilot study that assessed the dietary intake of patients with IBS or IBD who received FMT.^23^ Findings largely reflect individual dietary intake variability that is important to account for in human diet research.^8^ In addition, two case reports^28,29^ present findings in support of FMT with structured post-FMT diet therapy for the management of UC. Only one of the case reports^28^ included dietary intake data, which was reported to be ~ 1–1.2 g/kg/day protein and 25-30 g/day fiber per day. Two additional trials studied the use of adjunct diet therapy with FMT in patients with mild to moderate UC,^30,31^ and a single study assessed the impact of adjunct exclusive enteral nutrition therapy (EEN) in patients with active Crohn’s disease.^32^

**Table 3.** Overview of studies assessing post-FMT dietary intake.

Author	Year	Location	Design	Condition being treated	Intervention	Donor diet	Diet assessment method	Comments
Ge et al. ^24^	2016	China	Pilot study	Slow transit constipation	Fiber supplementation: Pectin, 8 g twice daily for 4 weeks	NA	NA	Daily log of GI symptoms was maintained Time-points of interest were 4- and 12-weeks post-FMT
Zhang et al. ^26^	2018	China	Prospective, Open-label	Slow transit constipation	Fiber supplementation: A Pectin, 8 g twice daily for unspecified time period (believed to be 12-months)	NA	NA	Similar design to Ge et al. Time-points of interest were 4-weeks and 1-year post-FMT
Costello et al. ^29^	2020	Australia	Case report	Ulcerative colitis	Dietary intervention: Increased fiber, reduced sulfate intake	NA	NA	19-year-old male Targeted diet therapy post-FMT
Bryant et al. ^28^	2021	Australia	Case Report	Ulcerative colitis	Dietary intervention: 4-SURE diet (similar to Costello et al.)	NA	7-d diet record at baseline and 3-d diet record at weeks 4, 8, and 24	71-year-old female Adherence to diet > 75% Post-FMT, protein intake of 1–1.2 g/kg/d and fiber intake of 25-30 g/d maintained until week 24
Clancy et al. ^23^	2021	Australia	Observational	Irritable bowel syndrome or Inflammatory bowel disease	None	NA	3-d diet records	Diet records collected at week 4, 12, and 24 post-FMT Fiber intakes ranged from 6-86 g/day (15-90 g/day if including supplements)
Koopen et al. ^35^	2021	The Netherlands	Double-blind, RCT	Obesity with Metabolic Syndrome	Mediterranean diet for 2-weeks pre-FMT. Continued for 6-weeks post-FMT.	NA	48-h diet record on 3 separate occasions	Subjects were provided food boxes suited for two adults to facilitate adherence. In total, subjects were placed on Mediterranean diet intervention for 8 weeks (−2 to 6-weeks post-FMT). This significantly changed intake. Diet 12-weeks post-FMT, however, no longer different from baseline.
Mocanu et al. ^36^	2021	Canada	Four-arm, parallel, placebo controlled, RCT Randomized 1:1:1:1 and stratified by sex	Obesity (BMI >30) with Metabolic syndrome	Fiber supplementation: Group 1: FMT+highly fermentable fiber Group 2: FMT+low fermentable fiber Group 3: Placebo FMT+highly fermentable fiber Group 4: Placebo FMT+low fermentable fiber	NA	Food frequency questionnaire	Fiber supplements taken over the 6-week course of the study Fiber supplement dosed at 33 g/day for men and 27 g/day for women Food frequency questionnaire used to assess dietary intake at baseline and week 6. No significant changes in dietary intake over the course of the study were observed
Xiang et. al. ^32^	2021	China	Randomized, prospective, open-label	Crohn’s Disease with Malnutrition	Exclusive enteral nutrition (EEN) intervention for 15 days + FMT on either day 1 or 8	NA	NA	Parenteral nutrition was used to achieve caloric intake of at least 30 kcal/kg/day EEN formula or amount of EEN not provided. Malnutrition diagnosed according to Patient-Generated Subjective Global Assessment
Huang et al. ^25^	2022	China	Two-arm, open-label, prospective	Refractory IBS-D	Dietary intervention: FMT or FMT+Diet Diet intervention: Low FODMAP diet	No fast food or alcohol^38^	Food diary	No dietary intake data provided. Only noted that participants were “required to maintain food diary”.
Kedia et al. ^30^	2022	India	Open-label, RCT	Ulcerative colitis	FMT+AID or Standard medical therapy	NA	7-d diet records or Food frequency questionnaire	7-d diet records and food frequency questionnaire were used interchangeably Dietary intake data collected at baseline and week 8. Dietary intake data difficult to interpret given reported median intake values of zero and no paired within-group pre-/post-FMT comparison
Shabat et al. ^31^	2022	Israel	Three-arm, single-blinded, RCT Randomized 1:1:1	Ulcerative Colitis	Group 1: FMT+Free diet Group 2: FMT+UCED diet Group 3: UCED diet	Group 1: NA Group 2: 2-week preconditioning with UCED diet prior to donating	NA	Diet adherence assessed, but methods not provided Dietary intake data not collected
Su et al. ^37^	2022	China	Open-label, RCT	Type 2 Diabetes	Fiber supplementation: FMT or FMT+Supplement Supplement: Probiotic, prebiotic, whole grain (PPW) mixture	NA	NA	No dietary intake data provided

RCT = Randomized Controlled Trial; NA = Not available; FODMAP = Fermentable Oligo-, Di-, Mono-saccharides, and Polyols; IBS-D = Irritable Bowel Syndrome with Diarrhea; UCED = Ulcerative Colitis Exclusion Diet; 4-SURE = 4-strategies-to-SUlfide-REduction diet; AID = Anti-Inflammatory Diet.

#### Post-FMT fiber supplementation studies

Four studies were identified that used an FMT plus fiber supplementation protocol.^24,26,36,37^ Two of the studies were in patients with slow transit constipation.^24,26^ In these two studies an isolated, singular, soluble fiber supplement (pectin) was used. Another study, in patients with obesity and metabolic syndrome,^36^ compared a highly fermentable fiber mixture against an isolated, singular, low fermentable fiber. The final study was in patients with type 2 diabetes mellitus (T2D)^37^ and used a complex supplement formula that consisted of a mix of different fibers along and a synbiotic powder. All four studies are limited by a lack of dietary intake data or fiber supplementation compliance/adherence data.

Two of the studies used an open-label, single-arm protocol of FMT with the addition of 16 g/day pectin supplementation for the treatment of slow transit constipation.^24,26^ The first study^24^ provides data on short-term (4-week and 12-week) outcomes with 4-week pectin supplementation. The second study^26^ provides long-term (12-month) results. Although not explicitly stated, pectin supplementation seemed to be over the entire year (along with a second and third FMT course as 1 and 3 months). No dietary intake data of donors or recipients was collected. Methods used to assess adherence to supplementation were not provided. Findings suggest that FMT with pectin supplementation may offer both short-term (4-week) and long-term (1-year) benefits to patients with slow transit constipation, but limitations in study design and data presented limit the ability to interpret findings.

A third study using a fiber supplementation protocol was a randomized, double-blind trial in patients with obesity (BMI >30) and metabolic syndrome.^36^ Participants were randomized 1:1:1:1 in blocks of four and stratified by sex into an FMT + high fermentable fiber, FMT + low fermentable fiber, Placebo FMT + high fermentable fiber, or Placebo FMT + low fermentable fiber cohort. Fiber supplementation was dosed at 33 g/day for men and 27 g/day for women. The high fermentable fiber consisted of soluble corn fiber, resistant wheat starch 4, and acacia gum. The low fermentable fiber was composed exclusively of microcrystalline cellulose. The primary endpoint of the study was a change in insulin sensitivity from baseline to week 6. Findings were in favor of the FMT + low fermentable fiber intervention, but there appears to have been a high degree of variability in response amongst participants. Notably, engraftment in the FMT + low fermentable fiber cohort was largely donor-mediated, with one specific donor having the highest engraftment of unique ASVs.

The fourth study was a 90-day, open-label trial assessing the effects of diet supplementation with or without FMT on gut microbiome and metabolic parameters in patients with T2D.^37^ The supplementation course consisted of three ready-to-consume foods: 1) 15 g mix of resistant dextrin, inulin, galactooligosaccharide, fructooligosaccharide, and xylooligosaccharide fiber powder; 2) 25 g organic wheat, oat, and highland barley whole grain powder mixture; and 3) a symbiotic powder preparation. Participants continued on their regular diets and consumed the supplement formulation three times per day. A total of 13 participants completed the study (supplement only *n* = 8; supplement+FMT *n* = 5). At 90 days, all participants in the study experienced weight loss, but statistically significant reductions in fasting blood glucose, glycated hemoglobin, and blood pressure were only observed in the supplement-only group. Dietary intake data or supplement adherence data was not collected or provided. Given the small sample size of both cohorts, there was limited evidence to form a definitive conclusion.

#### Post-FMT diet intervention studies

Five post-FMT diet intervention studies were identified.^25,30–32,35^ One in irritable bowel syndrome (IBS),^25^ two in UC,^30,31^ a single study in Crohn’s disease,^32^ and one in metabolic syndrome.^35^ Some of the studies included pre-FMT intake data and were discussed previously.^25,31,32,35^

The single study in an IBS population used an adjunct FMT+low FODMAP intervention for the treatment of IBS-D.^25^ This was an open-label trial in which participants were enrolled in an FMT only or FMT + low-FODMAP diet arm according to “whether or not study nutritionists felt they were suitable for the dietary intervention” based on 7-day diet records. The FMT-only cohort followed their baseline diet. The low-FODMAP cohort received a leaflet containing information on the low-FODMAP diet. While it is noted that patients were required to maintain a food diary, no dietary intake data is provided. As noted previously, donors with “good dietary habits” were chosen based on lack of fast food and alcohol consumption. IBS symptom severity scores and quality of life scores (IBS-SSS and IBS-QOL, respectively) were measured at baseline, 1-month, 3-months, and 6-months. Improvements in both IBS-SSS and IBS-QOL scores were observed in both cohorts, with instances of greater improvements observed in the FMT+low-FODMAP diet cohort. Findings are difficult to interpret, however, given the open-label study design and intentional groupings based on willingness to follow a low FODMAP diet. Additionally, without dietary intake data, it is unknown whether intake differed between the two cohorts. Ultimately, however, this study offers findings that suggest FMT is independently effective in treating IBS-D and that adjunct diet therapy may have an additive effect.

Two post-FMT diet studies were conducted in patients with mild to moderate UC. Both were published in 2022. One study^30^ used an anti-inflammatory diet (AID) intervention, and the other^31^ a UCED diet intervention (discussed above). In general, the AID diet intervention is a minimally processed diet focused on increased intake of fresh fruits, vegetables, and fermented foods and avoidance of a number of foods, including gluten-based grains, dairy products, and red meat.^30^ Subjects were randomized into two arms, an FMT+AID diet arm or standard medical therapy arm, and followed first for 8 weeks. At that time, only “responders” (decline in Simple Clinical Colitis Activity Index (SCCAI) by ≥ 3 points) were followed for an additional 40 weeks. Dietary intake data used was collected through either 7-day diet records or food frequency questionnaires if this was unavailable, but it is not reported what diet data were collected from which source. This type of information is important to consider because the selection of dietary assessment approaches depends on the intended use of the diet data.^43^ While dietary intake data comparisons at baseline, week 8, and week 48 are provided, interpretation is difficult as the categories in the FMT-AID group reduced at 8 weeks also trended lower than the standard medical therapy group at baseline. Adequacy of dietary intake on the FMT-AID diet was also a concern, with minimum daily calorie intake of 651 and 675 kcals and daily protein intake values of 24 and 18 grams at baseline and week 8, respectively. Additionally, clinical response was assessed as rates limited to those remaining in the study at any given time point. Finally, because the study design did not include an arm of exclusively diet or FMT, it is difficult to decipher whether or not diet may have had a complementary or independent effect on disease activity.

The second study in UC included three groups to assess the effects of FMT with adjunct diet therapy.^31^ Group 1 received FMT and followed their regular diet; Group 2 received diet preconditioned FMT from the same donors as Group 1 (donor stool was collected following a 2-week dietary conditioning period) and followed a defined ulcerative colitis exclusion diet (UCED); Group 3 followed the UCED diet alone. In general, the UCED diet intervention is a minimally processed, low-fat, moderate-to-high-fiber diet with an emphasis on specific fruits and vegetables. Participants assigned to one of the UCED diet arms received detailed instructions and a handout from a dietitian. The primary endpoint of this study was the intention to treat clinical remission determined by SCCAI score < 3 at Week 8 between Group 1 and Group 2. The trial was ultimately suspended due to futility. Notably, however, endoscopic remission at week 8 was found to be highest in the UCED diet-alone group (Group 3). Due to the loss to follow-up, however, compliance data at week 8 was only available for 8 of the original 15 participants in Group 3. While those participants were highly compliant (100%), if using noncompliance for missing data or withdrawals, this drops to ~ 50%. Methods for diet compliance assessment were not provided. In addition, dietary intake data was not available for this study, so it is difficult to assess how much diet changed or what impact this may have had on outcomes. Findings, however, suggest the ability to use post-FMT diet interventions in motivated patients with mild to moderate UC.

A single study was identified that assessed the use of adjunct exclusive enteral nutrition (EEN) therapy with FMT in patients with active Crohn’s disease (defined by Harvey-Bradshaw Index score > 4) and malnutrition.^32^ The study length was 15 days, and patients received EN through a nasogastric tube at a rate that provided a target energy intake of 30–35 kcal/kg per day (parenteral nutrition was added as needed to meet estimated calorie needs). Patients were only allowed water by mouth. Data suggests that FMT + EEN was more effective at improving nutrition status than EEN alone. While not included because it is a pediatric population, a retrospective analysis of pediatric patients with Crohn’s disease receiving either partial enteral nutrition (PEN) alone or PEN+FMT offers additional data suggesting an additive effect of PEN+FMT vs PEN alone in patients with Crohn’s disease.^44^

Finally, a single study was identified that studied adjunct diet therapy with FMT for the management of metabolic conditions.^35^ As discussed above, the study placed treatment naïve male subjects with obesity and metabolic syndrome on a Mediterranean diet for 2 weeks, at which time subjects were either randomized to receive lean donor or autologous FMT. Findings suggest a profound impact of 2-week diet therapy on study endpoints (microbiota composition and metabolic parameters) but limited additional effect of FMT. Notably, however, this study provided food boxes that included enough food for two adults so that meals did not have to be prepared separately, which is a robust method to support diet adherence. As noted above, 4-weeks after the diet phase ended (week 12), participants had returned to their baseline dietary intake patterns.

## Discussion

The landscape of FMT studies that include dietary intake data in humans support important nutritional considerations in both the pre- and post-FMT period. Studies highlight the potential role of diet in supporting compositional, functional, and clinical FMT endpoints. Although the available body of literature is relatively small and heterogenous, with limited descriptions of diet, this early work provides an important framework for researchers and clinicians to further study and apply diet therapy in FMT. The available body of literature also underscores, however, the need for future emphasis on collection of comprehensive and detailed diet data in future FMT studies.

### Compositional support

Two studies^20,35^ provide data demonstrating the sensitivity of human gut microbiome composition to short-term (2-week) diet changes in both stool donors^20^ and recipients.^35^ In the case of stool donors,^20^ a 2-week dietary preconditioning of donors – using the intended recipient therapeutic diet – led to a change in the relative abundance of 51 species and the depletion of nine microbial metabolic pathways. Further, the alignment of donor and recipient diet seemed to support metrics of improved engraftment compared to a comparator cohort where FMT material was from donors following their baseline diet and where recipients continued their baseline diet post-FMT. The work by Koopen et al. illustrates the impact of a 2-week Mediterranean pre-FMT diet intervention on microbial composition (increased abundance of *Bacteroides* species, *Akkermansia muciniphila*, *Roseburia hominis*, and reduced *Collinsella aerofaciens)*.^35^ An additional short-term (6-week) Mediterranean diet intervention with allogeneic or autologous FMT resulted in enrichment of unique donor-derived microbes in patients receiving allogeneic FMT compared to autologous FMT. The findings from these two studies suggest a role for both donor and recipient diet therapy to facilitate engraftment of microbes of interest in FMT trials.

A fiber supplementation study in patients with obesity (BMI >30) and metabolic syndrome^36^ points out the role long-term dietary intake of the donor may play in response to a post-FMT intervention. In this case, engraftment in the FMT + low fermentable fiber cohort was largely donor-mediated, with one specific donor having the highest engraftment of unique ASVs. This finding is likely due to a need for the presence of specific taxa to ferment the low fermentable microcrystalline cellulose^45^ compared to the highly fermentable fiber, which is more widely used by the gut microbes.^46^ In instances such as this, where specific donor taxa may be required to utilize the supplemented product, knowledge of long-term donor dietary intake may allow for a more informed donor selection process in future trials.

### Functional support

The work by Smits et al.^27^ illustrates the importance of dietary intake to microbiome function and the potential importance of aligning donor and recipient dietary intake. The researchers found no change in TMAO production with FMT product from vegan donors in patients with metabolic syndrome even though a compositional shift toward vegan donor microbiome in recipients occurred. These findings reflect what was likely suppressed TMAO production in the vegan donors compared to recipients resulting, in part, from increased fiber intake (43 g/day vs 18 g/day, respectively). Dietary fiber is capable of suppressing microbial TMAO production.^47^ Therefore, the lack of change in TMAO production in the FMT recipients, in the context of donor-driven compositional microbiota changes, may simply have been the result of reduced dietary fiber intake.

Future studies should consider aligning post-FMT recipient intake with donor dietary intake or selecting stool donors with functional features of interest, such as low TMAO-production, who have a similar dietary intake to recipients. The alignment of donors with dietary intake similar to recipients, when possible, is likely to be the most effective long-term approach. The foods we eat are informed by much more than intentional selection^48^ making it difficult to facilitate long-term changes to an individuals’ habitual dietary intake. Knowing this, it would be prudent to screen and select FMT donors who harbor microbiome characteristics of interest and are also following a diet similar to most recipients. Alternatively, rather than aligning donor and recipient dietary intakes, selection of FMT donors could involve challenging the donor microbiome with the anticipated recipient diet – vs therapeutic diet^31^ – to determine if the desired microbiome features are likely to be maintained (e.g., TMAO production) on the recipient diet.

### Clinical support

Ensuring patients receive adequate nutrition prior to undergoing FMT may help augment treatment outcomes, particularly in conditions associated with a high amount of inflammation and metabolic stress, such as rCDI or IBD. In the case of rCDI, malnutrition is highly prevalent,^12,39^ and pre-FMT zinc status was shown to influence the success of therapy.^22^ Also of clinical note is the finding that FMT seemed to provide a synergistic effect along with nutrition therapy to support the nutritional recovery of patients with active CD.^32^ Perioperative nutrition, including both dietary preconditioning and post-transplant nutrition, is increasingly understood to play an important role in the success of solid organ transplantation.^49^ Approaching dietary management of patients at risk for malnutrition undergoing FMT through a similar lens may allow clinicians and researchers to optimize the success of FMT treatment in both clinical and research settings.

In addition to illustrating the effect of short-term diet on microbiome composition, the work by Koopen et al.^35^ also underscores the pronounced, independent effect of diet therapy on clinical outcomes such as metabolic parameters in patients with metabolic syndrome (pre-FMT).^50^ In this study, 2-weeks of Mediterranean diet therapy pre-FMT led to reductions in body weight, insulin resistance, and lipid levels. In the 6-weeks following FMT, no additional benefit on metabolic parameters of interest were observed. These findings underscore the independent therapeutic effect of diet on metabolic endpoints and its potential role as an adjunct to FMT. A number of studies included in this review do suggest an additive role for diet therapy on clinical FMT outcomes with studies supportive of FMT with fiber supplementation to manage constipation, FMT with diet therapy in mild to moderate UC, and FMT with a low FODMAP intervention to manage IBS. However, the role of adjunctive diet therapy in FMT requires further research as methodological limitations within the reviewed studies limits interpretation of findings.

### Considerations for future research

The impact of pre- and post-FMT nutrition on the success of therapy remains unclear, but the available literature highlights the need for the adoption of more robust diet assessment methods. Given that microbiome composition reflects some aspects of habitual diet and other personal factors, it may be important to consider the impact of diet on both the donor and recipient to better understand the dietary features that support success of FMT. Dietary assessment methodology in the context of human gut microbiome research has been reviewed and summarized previously.^51,52^ Briefly, there are three predominant types of dietary collection methods that rely on participant self-report. Food frequency questionnaires provide estimates of habitual dietary intake and ask participants to report their usual intake over a given time period (e.g., the previous 30 days or the past 12 months). Food diaries or food records are collected in real-time by participants and provide information about intake over 1 or multiple days, with dietary details collected throughout the recording period by the participant using electronic or paper records. 24-hour recalls are typically researcher administered and ask participants to recall their intake for the prior 24-hour period. Automated 24-hour recalls are available and can be used to reduce researcher burden such as the Automated Self-Administered 24-hour (ASA24) Dietary Assessment Tool, developed by the National Cancer Institute.^53^ When selecting a method for dietary data collection, it is essential for researchers to consider at the study onset how they intend to use their dietary data to ensure that the variables of interest will be available. For example, certain dietary data collection methods allow for the analysis of subclasses of fiber, such as insoluble and soluble fiber, and pectin^54^ while other databases provide one variable of fiber and do not disaggregate fiber.^55^

Traditional dietary assessment methodology has the goal of identifying the essential energy and nutrient intake of the host. However, because the microbiome uses the end-products of human digestion for fermentation and may primarily metabolize the non-nutritive components of diet that have not been traditionally well documented in nutritional databases, there are significant challenges when aiming to connect diet (using traditional dietary assessment methodology) and microbiome.^56^ Using foods and food groups to derive dietary patterns may improve the ability to assess the impact of diet on gut microbiome composition as a result.^56,57^ This approach may also help account for altered nutrient flow to the gut microbes based on the degree of food processing. Food processing can alter the physical functions of fiber,^58^ and critically, as it pertains to the gut microbiota, change the quantity of microbiota-accessible carbohydrates reaching the colon^59–62^ and rate of fermentation.^63–65^ What this means in practice is that 10 g of dietary fiber from a whole food source will be presented to and fermented differently by the gut microbiota than 10 g of the same fiber in a purified and isolated form. Conceptually this is similar to well established phenomena in human digestion where degree of processing (e.g. whole peanuts vs peanut butter vs peanut oil) impacts calories and nutrients absorbed.^66^ Improved analysis methods^56,57^ coupled with advancements in dietary intake assessment technologies will help address what remains a major challenge to diet-microbiome research.

While the diet assessments used in prior studies provide useful information, there is a paramount need to accurately capture acute and chronic diet variations that impact the gut microbiome. Recent technological advances have examined several objective approaches that can be used to determine dietary intake, including image-based sensing, eating action unit sensors, biochemical measures or biomarkers, and sequence-based technologies – all which require further testing and evaluation.^67^ The future generation of diet assessments is moving toward techniques that will lessen participant burden through passive sensing, while providing just-in-time information about one’s diet.^68^ The focus on just-in-time capture of intake will be useful to investigate the immediate effects of how one’s diet, meal timing, and eating behaviors can influence gut microbiome remodeling in granular detail.^69^ Further, to fully understand the impact that diet can have on the gut, it is essential to collect information about how contextual factors (i.e., environment, stress) interact with one’s dietary intake to alter the gut microbiome.^70^ These interactions remain largely unexplored and future research is needed to investigate factors that modify the associations between diet and gut health.

## Conclusion

FMT has emerged as a promising therapeutic approach for various disease states, but it requires continued optimization. Diet is a modifiable variable impacting composition and function of the gut microbiome that can be harnessed to improve success of FMT. Exploring the effect of pre- and/or post-dietary intake on FMT-associated outcomes in humans represents a critical avenue of research with profound implications for clinical practice and therapeutic optimization. As part of this effort, there should also be a focus on using validated approaches to collect detailed diet data, including processing methods, dietary patterns, and environmental interactions. By considering the intricate interplay between diet, gut microbiota, and FMT efficacy, we may be able to develop microbiome-targeted precision nutrition strategies that advance the paradigm of FMT and clinical nutrition and, ultimately, improve patient outcomes.

## Data Availability

Data sharing is not applicable to this article as no new data were created or analyzed in this study

## Appendix A: Search Strategies

All searches as they were run on May 13, 2024.

### Medline via Ovid

1. exp Fecal Microbiota Transplantation/
2. ((Fecal or Faecal or microbiota or microflora or feces or feces or stool) adj3 (transplant* or transfus* or implant* or instillation or donor* or enema or reconstitution or infusion* or therap* or transfer* or treat*)).tw.
3. ((bacteria or bacterio*) adj2 (transplant* or transfus* or implant* or instillation or donor* or enema or reconstitution or infusion* or therap* or transfer* or treat*)).tw.
4. (bacteriotherap* or colonic restoration or flora reconstitution).tw.
5. FMT.ab.
6. or/1-5
7. nutrients/
8. exp Nutritional Physiological Phenomena/
9. (nutrient* or nutrition* or food* or diet* or supplement*).tw.
10. or/7-9
11. 6 and 10
12. exp animals/not humans.sh.
13. 11 not 12

### Agricola via Ovid

1. ((Fecal or Faecal or microbiota or microflora or feces or feces or stool) adj3 (transplant* or transfus* or implant* or instillation or donor* or enema or reconstitution or infusion* or therap* or transfer* or treat*)).mp.
2. ((bacteria or bacterio*) adj2 (transplant* or transfus* or implant* or instillation or donor* or enema or reconstitution or infusion* or therap* or transfer* or treat*)).mp.
3. (bacteriotherap* or colonic restoration or flora reconstitution).mp.
4. FMT.ab.
5. or/1-4
6. nutrients/
7. exp nutrition/
8. (nutrient* or nutrition* or food* or diet* or supplement*).tw.
9. or/6-8
10. 5 and 9
11. mice.ab.
12. human.ab.
13. 11 not 12
14. 10 not 13

### Embase via Ovid

1. fecal microbiota transplantation/
2. ((Fecal or Faecal or microbiota or microflora or feces or feces or stool) adj3 (transplant* or transfus* or implant* or instillation or donor* or enema or reconstitution or infusion* or therap* or transfer* or treat*)).tw.
3. ((bacteria or bacterio*) adj2 (transplant* or transfus* or implant* or instillation or donor* or enema or reconstitution or infusion* or therap* or transfer* or treat*)).tw.
4. (bacteriotherap* or colonic restoration or flora reconstitution).tw.
5. FMT.ab.
6. or/1-5
7. exp nutrition/
8. (nutrient* or nutrition* or food* or diet* or supplement*).tw.
9. 7 or 8
10. 6 and 9
11. (exp animal/or nonhuman/) not human/
12. 10 not 11
13. limit 12 to exclude medline journals

### Cochrane Library via Wiley

#1MeSH descriptor: [Fecal Microbiota Transplantation] explode all trees

#2((Fecal or Faecal or microbiota or microflora or feces or feces or stool) NEAR/3 (transplant* or transfus* or implant* or instillation or donor* or enema or reconstitution or infusion* or therap* or transfer* or treat*)):ti,ab,kw

#3((bacteria or bacterio*) NEAR/2 (transplant* or transfus* or implant* or instillation or donor* or enema or reconstitution or infusion* or therap* or transfer* or treat*)):ti,ab,kw

#4(bacteriotherap* OR “colonic restoration” OR “flora reconstitution”):ti,ab,kw

#5(FMT):ab

#6#1 OR #2 OR #3 OR #4 OR #5

#7MeSH descriptor: [Nutrients] this term only

#8MeSH descriptor: [Nutritional Physiological Phenomena] explode all trees

#9(nutrient* or nutrition* or food* or diet* or supplement*):ti,ab,kw

#10#7 OR #8 OR #9

#11#6 AND #10

### Scopus via Elsevier

(TITLE-ABS-KEY (nutrient* OR nutrition* OR food* OR diet* OR supplement*)) AND ((TITLE-ABS-KEY (fecal OR fecal OR microbiota OR microflora OR feces OR feces OR stool) W/3 TITLE-ABS-KEY (transplant* OR transfus* OR implant* OR instillation OR donor* OR enema OR reconstitution OR infusion* OR therap* OR transfer* OR treat*)) OR (TITLE-ABS-KEY (bacteria OR bacterio*) W/2 TITLE-ABS-KEY (transplant* OR transfus* OR implant* OR instillation OR donor* OR enema OR reconstitution OR infusion* OR therap* OR transfer* OR treat*)) OR (TITLE-ABS-KEY (bacteriotherap* OR “colonic restoration” OR “flora reconstitution”)) OR (ABS (fmt))) AND NOT INDEX (medline) AND (LIMIT-TO (SUBJAREA, “MEDI”) OR LIMIT-TO (SUBJAREA, “IMMU”) OR LIMIT-TO (SUBJAREA, “HEAL”))
